# A multi-site, multi-modal travelling-heads resource for brain MRI harmonisation

**DOI:** 10.1038/s41597-025-04822-2

**Published:** 2025-04-11

**Authors:** Shaun Warrington, Andrea Torchi, Olivier Mougin, Jon Campbell, Asante Ntata, Martin Craig, Stephania Assimopoulos, Fidel Alfaro-Almagro, Karla L. Miller, Mark Jenkinson, Paul S. Morgan, Stamatios N. Sotiropoulos

**Affiliations:** 1 Sir Peter Mansfield Imaging Centre, Mental Health and Clinical Neurosciences, School of Medicine, University of Nottingham, Nottingham, UK; 2 Sir Peter Mansfield Imaging Centre, School of Physics, University of Nottingham, Nottingham, UK; 3 Wellcome Centre for Integrative Neuroimaging, FMRIB Centre, Nuffield Department of Clinical Neurosciences, University of Oxford, Oxford, UK; 4 National Physical Laboratory, Teddington, Middlesex UK; 5 Australian Institute for Machine Learning (AIML), School of Computer and Mathematical Sciences, The University of Adelaide, Adelaide, Australia; 6 South Australian Health and Medical Research Institute (SAHMRI), Adelaide, Australia; 7 Nottingham NIHR Biomedical Research Centre, Nottingham University Hospitals NHS Trust, Queen’s Medical Centre, Nottingham, UK

**Keywords:** Neuroscience, Magnetic resonance imaging

## Abstract

Despite its great potential for studying the living brain, magnetic resonance imaging (MRI) can be often limited by nuisance non-biological factors, such as hardware/software differences between scanners, which can interfere with biological variability. This lack of standardisation or harmonisation between scanners hinders reproducibility and quantifiability of MRI. Towards addressing this challenge, we present one of the most comprehensive MRI harmonisation resources, based on a travelling heads paradigm; healthy volunteers scanned repeatedly across different scanners. The Oxford-Nottingham Harmonisation (ON-Harmony) resource offers data from 20 participants each scanned on six different 3 T MRI scanners from three major vendors (GE/Philips/Siemens) across five imaging sites. Each scanning session includes five imaging modalities (T1w/T2w/dMRI/rfMRI/SWI) with protocols aligned to the UK Biobank, while for about half of the participants five within-scanner repeats are additionally acquired. The 165 multi-modal scanning sessions allow mapping of different pools of variability (biological, between-scanner, within-scanner) for hundreds of MRI-derived measures. We describe the breadth of information contained in the publicly-available data and showcase their reuse potential for evaluating efficacy of harmonisation approaches.

## Background & Summary

A unique strength of magnetic resonance imaging (MRI) of the brain is the ability to obtain a range of multi-modal measurements, reflecting different aspects of neuronal tissue structure, microstructure, function, physiology and connectivity^1–3^. However, measurements are hampered by the lack of standardisation or harmonisation across scanners^4,5^. Consistency in measuring the same individual across multiple MRI devices is challenged by nuisance, non-biological sources of variability^6,7^. Differences in scanner hardware, software, scanning protocols, operators, and image processing methods can all affect imaging-derived measurements in non-trivial ways, reducing their quantifiability. Induced between-scanner variability for an individual has been found in cases to be even comparable to biological between-participant variability^8–10^, while consistency of participant ranking based on MRI measurements can also be affected across scanners^9^. MRI harmonisation efforts are therefore crucial for improving the reproducibility of results across experiments, facilitating meaningful comparisons between datasets, enabling reliable longitudinal research, and combining data effectively to increase sample sizes. This lack of reproducibility also limits the application of MRI in clinical research and/or clinical trials^11^.

A number of approaches have been proposed for MRI harmonisation. As a first step, harmonising the scanning protocols across scanners can mitigate some of these issues, however, it is often only partially effective, due to vendor-specific implementations and restrictions. Differences in hardware can make parameter matching difficult or impossible, and even nominally matching scanning parameters does not always result in true consistency. A new framework, Pulseq.^12^, aims to remove a level of such protocol (and hardware) variability using vendor-agnostic protocols, which can even match scanners to the lowest denominator in terms of hardware. Between-scanner acquisitions of the same individuals using Pulseq protocols have been shown to be more consistent than vendor-specific acquisitions across a number of modalities^13–15^. Despite the great potential, it is still unclear to what degree such efforts will fully resolve the challenges, as they currently are non-trivial to implement (e.g. reliant on bespoke offline image reconstruction).

Other approaches for harmonisation use post-processing algorithms either on the MRI signal^8,16^ or on the imaging-derived features^17,18^. For example, the commonly-used ComBat algorithm, originally developed for microarray data^19^ and now extended to MRI data^17,20^, models batch effects as additive and multiplicative factors and adjusts the mean and variance of extracted features accordingly; whilst avoiding the removal of biological variance by considering participant age and sex as covariates. There are now several tools that extend on these principles to remove batch effects from extracted measures, employing more advanced modelling of batch effects and/or targeting specific imaging modalities^6,18,21^.

A challenge in all proposed solutions is evaluating harmonisation efficacy. Some studies have employed methods based on participant group matching, using categories such as age, sex, ethnicity, and handedness^18,20^ to attempt to regress out scanner-related differences. Other studies assess efficacy by the degree to which harmonised data no longer inform on the scanner they originated from (scanner “unlearning”)^22–24^. A more direct, though complex and not always feasible, approach involves acquiring images from *travelling heads*. The same participants can be repeatedly scanned across different scanners in order to understand scanner/batch effects. Within-participant-between-scanner differences can then be compared against a scan-rescan baseline, where the same participant is scanned under identical conditions (protocol, and processing) in the same scanner (within-participant-within-scanner variability). Such studies can provide insight into how different imaging features are sensitive to between-scanner effects, be used to develop generalisable processing pipelines and act as testbeds for developed harmonisation frameworks^9^.

Here, we present one of the most comprehensive travelling-heads studies performed to date for multi-modal MRI of the brain. The Oxford-Nottingham harmonisation (ON-Harmony) resource comprises more than 160 multi-modal brain MRI scan sessions, obtained from 20 healthy individuals. These 20 participants were each scanned in six different 3 T MRI scanners (from a pool of eight MRI scanners considered in total), located at five different imaging sites in Nottingham and Oxford in the UK, and with five imaging modalities: T1-weighted (T1w), T2-weighted (T2w), susceptibility-weighted imaging. (SWI), diffusion MRI (dMRI), and resting-state functional MRI (rfMRI). The scanners cover three major vendors (Siemens, Philips, GE) and different generations of scanners from these vendors. For nine of the participants, an additional five rescans were obtained in the same scanner (with different scanners chosen for different participants), allowing characterisation of between-scanner, within-scanner and between-participant variability. We previously presented an early subset of this study (Phase A, 10 participants ∼45% of the data)^9^. Here, we present the data resource in its entirety, showcasing data with better balance in the considered scanner vendors, in participant sex and lower inter-scan delays during the latest phase (Phase B) of data acquisition. We also provide technical validation of the data, by considering the two independent cohorts (Phase A and B) and showcasing reproducibility of patterns. Compared to a number of previous travelling heads studies^7,10,25–34^, this resource combines more neuroimaging modalities, in total up to eight scanners from all three vendors, whilst including scan-rescan repeats for establishing within-participant within-scanner baselines.

In this article, we describe the experimental setup, participant demographics and acquisition protocols of ON-Harmony and perform quality control of the acquired data for imaging artefacts, distortions, noise and motion. We use a variant of the UK Biobank (UKBB) processing pipeline^35,36^ to extract hundreds of multi-modal imaging features for each scanning session and present different pools of variability for these features (between and within-scanner for the same participant, as well as between-participant), showcasing the breadth of information conveyed by this data. We highlight benefits from the design improvements in Phase B of the acquisition, compared to the previously published Phase A of the data^9^, as well as generalisability of reported trends across the two phases. We indirectly validate this resource further and demonstrate its reuse potential by showing how post-processing harmonisation can mitigate the gap of between-scanner and within-scanner sessions.

ON-Harmony is released in BIDS standard format^37^ via OpenNeuro (doi:10.18112/openneuro.ds004712.v2.0.1)^38^, allowing simple and open access. We anticipate it will be a valuable resource for development and assessment of new *explicit harmonisation* algorithms. Harmonisation methods are typically assessed by the removal of associations between imaging features and scanner/site, whilst retaining subject demographics^18,20,22^. ON-Harmony offers a complementary means for evaluating harmonisation techniques by providing reference baselines for different sources of variability. In addition, the resource enables the development, optimisation and testing of vendor-agnostic image processing pipelines (*implicit harmonisation*), which also contribute to variability and bias of imaging-derived features^9,39–41^. For instance, tools that have been developed and tested using data from certain vendors only, can have significantly less generalisability when applied to data from other vendors. ON-Harmony can close this gap by acting as a test-bed for developing generalisable tools and pipelines. Finally, even if not aimed and suited for vendor or scanner performance comparison, ON-Harmony data also reflects how well-aligned the acquisition protocols that are used here are across a range of scanners, and which imaging-derived features are more/less reliable than others.

## Methods

### Study design

**Fig. 1:**
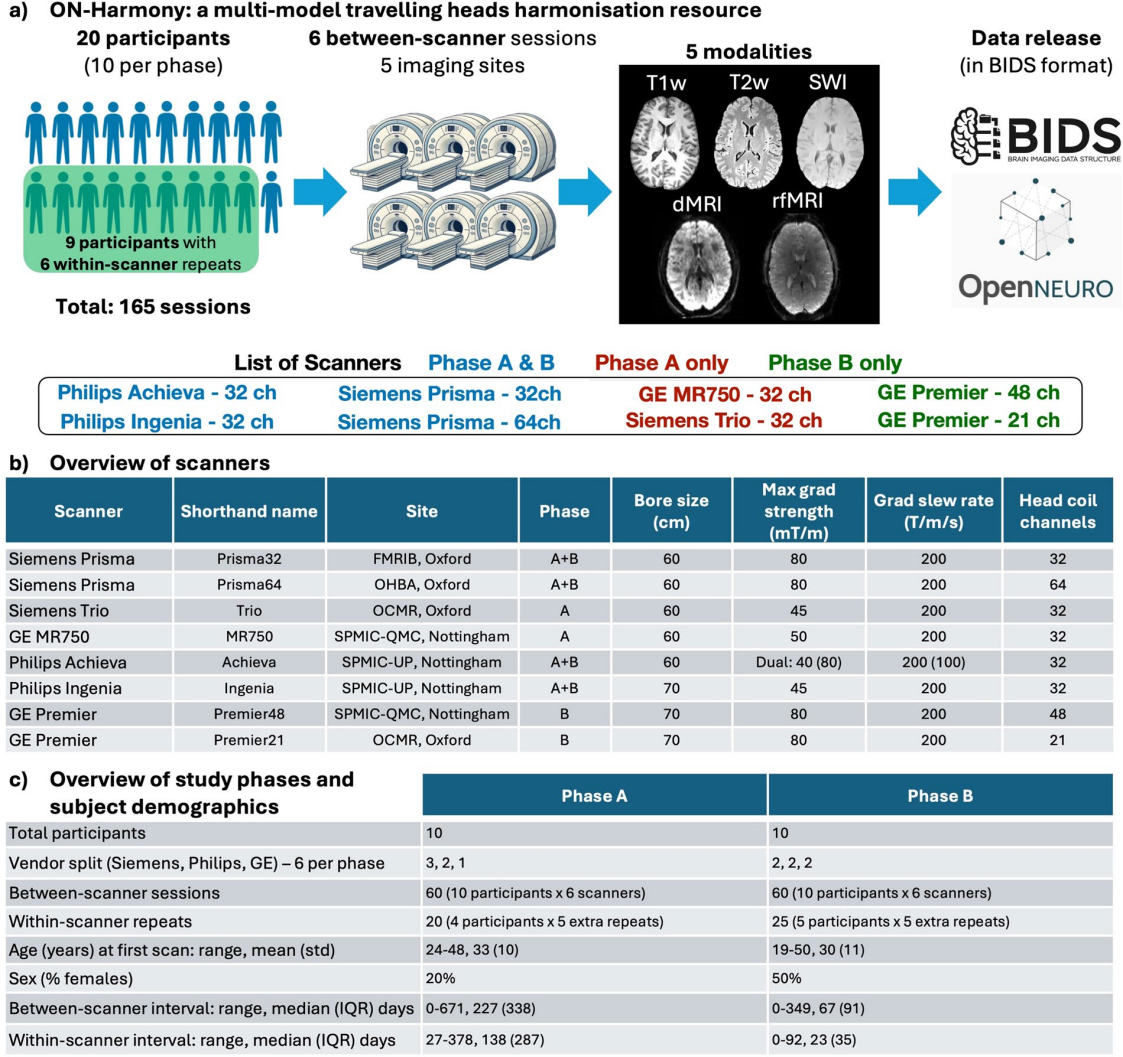
A schematic overview of the ON-Harmony resource and summaries of scanner details and participant demographics. (**a**) ON-Harmony is a multi-modal harmonisation resource consisting of 20 travelling-heads each scanned on six different 3 T scanners across the three major MRI vendors across two phases, with nine participants completing an additional five within-scanner repeat scans. For each session, five imaging modalities are acquired: T1w, T2w, SWI, fMRI and dMRI. All data are converted to BIDS format and released via OpenNeuro. (**b**) An overview of the included scanners and their key specifications. (**c**) A summary of participant demographics and study design split by phase.

Twenty healthy participants (31.5 (10.5) years old mean (standard deviation) age, seven females) had their brains scanned, each in six different 3 Tesla (T) MRI scanners with five neuroimaging modalities (T1w, T2w, SWI, dMRI, rfMRI). Scanners were located in Nottingham and Oxford at five different imaging sites: the Sir Peter Mansfield Imaging Centre University Park (SPMIC-UP) and Sir Peter Mansfield Imaging Centre Queen’s Medical Centre Medical School (SPMIC-QMC) in Nottingham, the Oxford Centre for Functional MRI of the brain (FMRIB), the Oxford Centre for Human Brain Activity (OHBA) and the Oxford Centre of Magnetic Resonance (OCMR). Figure 1a shows an overview of the study design. Data were acquired in two phases with 10 participants in each phase and four scanners being used across both phases, with two scanners changing from Phase A to Phase B (Fig. 1b). Phase A took slightly more than two years to complete, as it occurred within the Covid-19 pandemic. Phase B was uninterrupted and completed in six months, leading to significant reductions in the between-session intervals per participant (both for between-scanner and within-scanner repeats, Fig. 1c). We also achieved a better balance in the sex of the participants and the split of scanners across vendors for Phase B compared to Phase A.

Figure 1b summarises the 3 T scanners used, which covered three major vendors (Siemens, GE and Philips). Hardware differences included, amongst others, different bore sizes, maximum gradient strengths and number of channels in the head coil. In each scanning session, five neuroimaging modalities were acquired covering structural, microstructural and functional MRI. Each of the 20 participants had six between-scanner sessions, while nine of them (four in Phase A and five in Phase B) had six within-scanner repeats, totalling 165 multi-modal scanning sessions.

### Participant recruitment

Participants were recruited through posters displayed on approved notice boards around the University of Nottingham and University of Oxford, word-of-mouth and emails to approved departmental mailing lists. For inclusion in the study, apparently healthy adults, 18–55 years old were considered, who were able and willing to give informed consent and had no symptoms nor were they taking any medications (if they were on long-term medication for a non-neurological/non-psychiatric condition (e.g. inhalers) and were stable with no symptoms, they were also considered for this study). Exclusion criteria were as follows: inability to complete MRI safety questionnaire and/or informed consent process; known contraindication to MRI scanning (for example pacemaker/implanted defibrillator, intracranial vascular clip, implanted programmable device, intra-ocular metallic fragment, etc); current or previous neurological, neurosurgical, psychiatric, cognitive or mood disorder; any other significant chronic medical condition; claustrophobia; pregnancy; cranioplasty, craniofacial reconstruction, fixed dental brace or other craniofacial metalwork that is likely to cause significant image degradation or artefact; inability to travel to the imaging centres in Nottingham and Oxford. The first ten participants to meet these criterion for each phase were included in the study. Immediately prior to each MRI session, participants underwent detailed MRI safety screening as per local protocols.

### Data acquisition

For the MRI acquisition protocols of all five neuroimaging modalities, we used the UKBB^35^ imaging protocol as a guide. This is a relatively short multi-modal protocol (about 35 minutes in total) developed originally for Siemens scanners. We adapted the protocol to all scanners used in this study. We matched acquisition parameters as close as possible to the UKBB protocol, while respecting best practices and limitations for each scanner and imaging site. This resulted in aligned protocols that provided a middle-ground solution between direct matching acquisition parameters and having comparable image quality, as nominal matching of protocol parameters across scanners and vendors can lead to compromised data quality^9^.

**Fig. 2:**
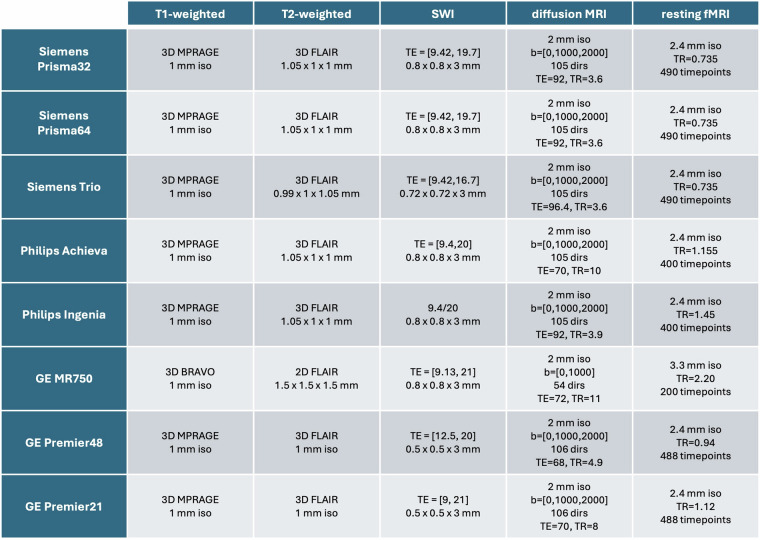
Summary of key acquisition protocols across all modalities and scanners. TR is reported in seconds. TE is reported in ms.

Figure 2 provides a summary of the main acquisition parameters, across the five modalities and the scanners used throughout the study. To aid consistent acquisition, scanner operators were provided with standard operating procedures to guide acquisition. The protocol descriptions below for scanners common across phases have been previously described^9^. We reproduce them here, for completeness, along with descriptions of protocols for the newly added scanners (GE Premier48 and GE Premier21).

High-resolution T1w gradient echo (3D MPRAGE)^42^ were acquired for all scanners with 1 mm isotropic voxel size, apart from the MR750 where a 3D BRAVO sequence was used. Gradient non-linearity distortion correction (GDC) was turned off for the Siemens scanners because the Siemens on-scanner corrections have been found to provide inconsistent results, particularly for 2D echo planar imaging (EPI) acquisitions^36^. For the non-Siemens scanners, GDC correction was performed on the scanner. This applies to all other modalities we acquired. Vendor-provided pre-scan normalise was used for all scanners.

A 3D T2w FLAIR, with close to 1 mm isotropic voxels, was also acquired, with the exception of the MR750 that did not have that 3D capability. Instead, we obtained a 3D FLAIR without T2w for that scanner and also acquired 2D T2w FLAIRs, which are inherently slower than 3D and compromised spatial resolution. In total, we acquired three versions: (i) 1 mm isotropic 3D FLAIR, (ii) 1.5 mm isotropic 2D T2w FLAIR, and (iii) 1 × 1 × 2 mm 2D T2w FLAIR. The same GDC and pre-scan normalise options were followed as before.

Multi-shell pulsed gradient spin echo (PGSE) diffusion MRI (b = 1000 & 2000 *s/mm*^2^) was acquired in all scanners, with the exception of the MR750, where only a single b = 1000 *s/mm*^2^ shell was acquired. Blip-reversed spin-echo b = 0 volumes were also acquired for each session to correct for susceptibility-induced distortions^43^, with the phase-encoding direction switching along the anterior-posterior orientation. Differences in gradient strength and multiband acceleration capabilities affected the minimum achievable echo time (TE) and repetition time (TR) across scanners. Both the Achieva and MR750 did not have multiband capabilities, therefore the resulting TR was above 10 seconds. For the Premier48, we used GE’s ABCD patch, which allowed lower TR with a multiband factor of two, due to a different thermal heating model. For the Premier21, we used the product dMRI sequence, which had a longer TR. Spatial and angular resolution across b-shells was matched for all scanners (2 mm isotropic voxels and ∼50 volumes per shell).

For resting-state fMRI, we used a 2D gradient echo EPI sequence. Participants were asked to keep their eyes open and think nothing in particular. We aimed to be close to 1 second TR and for a relatively high spatial resolution (2.4 mm isotropic), which was possible for most scanners, apart from the MR750, where we had to compromise both in spatial and temporal resolution. The longer TR resulted in approximately half the number of time points (200 compared to at least 400 volumes for all other scanners). Deviations between scanners were due to differences in acceleration and hardware capabilities. In particular, for the Premier21, a lower multiband factor was used as the number of channels in the head coil was considerably lower compared to the other scanners. In each scanner and protocol, the flip angle was set to the Ernst angle for the corresponding TR, assuming longitudinal relaxation time (T1) of 1.5 seconds for grey matter at 3 T.

Finally, for SWI, two echoes were obtained at roughly the same echo times (*TE*_1_ = 9 and *TE*_2_ = 20 milliseconds) and complex data (magnitude and phase) were saved. For the GE scanners, the SWAN sequence was used, which acquired seven echoes, and the two echoes closer to *TE*_1_ and *TE*_2_ were extracted. This resulted in a higher bandwidth for the GE data compared to Philips and Siemens. Accurate reconstruction of phase images required the complex sensitivity of the individual coil data as anomalous phase transitions in regions of focal dropout have been reported^36,44^. For the Siemens scanners, data from individual coils were saved separately, and phase images were subsequently high-pass filtered and combined during post-processing. For the non-Siemens scanners, such anomalous phase transitions are less common^36,44^ and hence individual coil data were combined on the scanner.

**Fig. 3:**
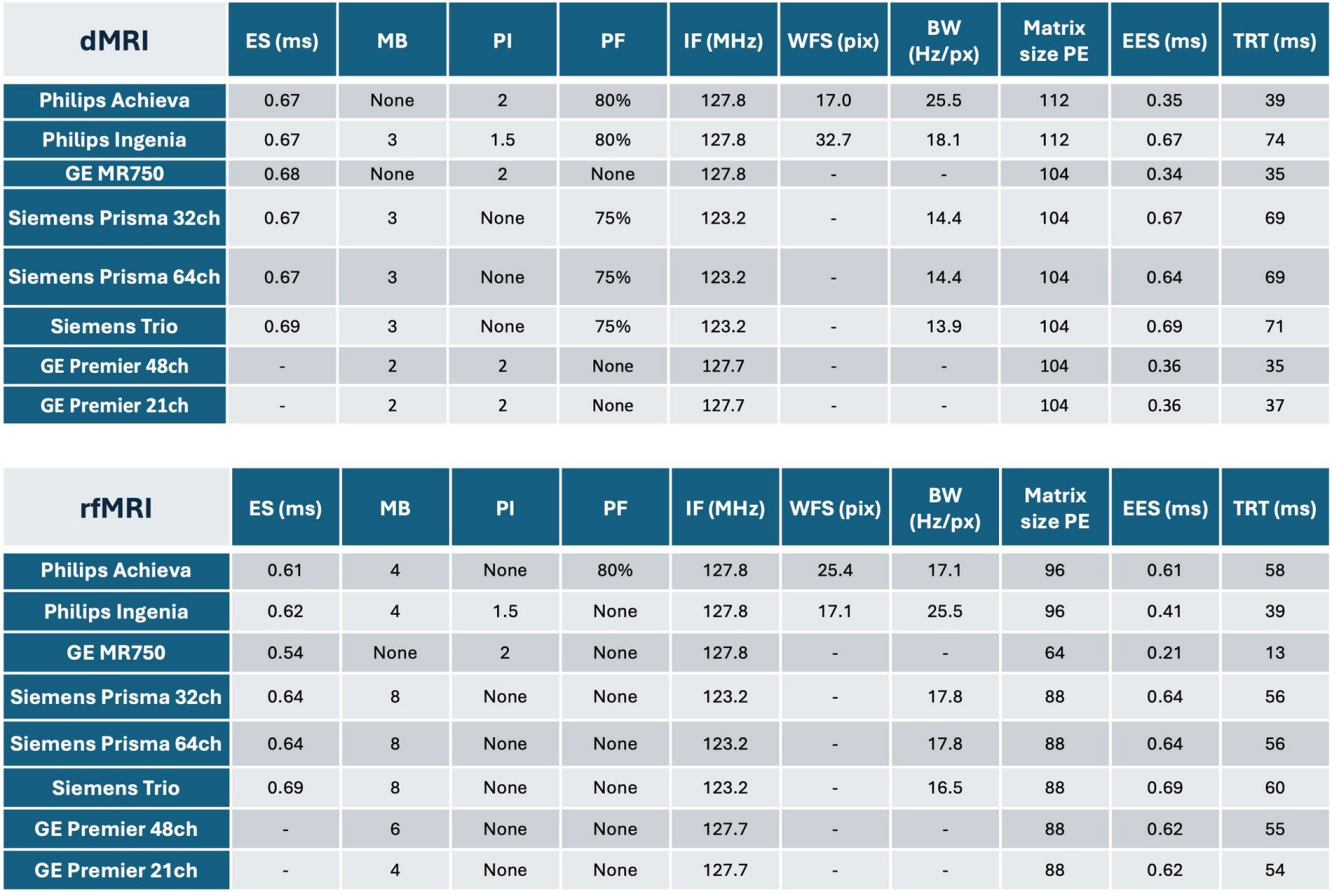
Effective echo spacing (EES) and total readout times (TRT) for dMRI (top) and fMRI (bottom) used in distortion corrections. Values displayed are as reported from dcm2niix (v1.0.20211006). ES = echo spacing; MB = multiband factor; PI = in-plane acceleration factor; PF = partial Fourier; IF = imaging frequency; WFS = water fat shift (pixels); BW = phase encoding bandwidth per pixel (Hz/pixel); Matrix size PE = image (acquisition and reconstruction) matrix size in the phase encoding direction; EES = effective echo spacing; TRT = total readout time. Unavailable parameters are denoted by ‘-’. Adapted from^9^ and augmented with new scanners.

Details of in-plane and out-of-plane accelerations used for each scanner for the dMRI and rfMRI protocols are summarised in Fig. 3. We also report parameters for the calculation of the effective echo spacing (EES) and total readout time (TRT), which can be used for susceptibility-induced distortion correction. These parameters were extracted from dcm2niix (v1.0.20211006)^45^ and following the formulas summarised previously^9^, which take into account nominal echo-spacing, in-plane acceleration, as well as bandwidth per pixel and matrix dimensions.

Shimming was performed at the beginning of each session and auto-reshimming during the session was disabled. Final protocol parameters were also chosen so that the total scan time per session did not exceed an hour (with the Achieva and MR750 having the longest sessions, due to lower acceleration capabilities). Scanner interpolation was switched off for all sessions and data were reconstructed at the acquired spatial resolution.

Scanner software version was maintained for the majority of sessions where possible. Prisma scanners used software version Syngo MR E11 and the Trio scanner used Syngo B17. The MR750 used software version DV 24.0., while the Premier scanners used software version DV 29.1. For the Philips scanners, the software version was upgraded during the study. For the Achieva, six participants were acquired with version 5.3.0.3, while all other 14 participants were acquired with version 5.6.1.1. For the Ingenia, six participants were acquired with version 5.3.1.0, 13 participants were acquired with version 5.6.1.0, and one participant (with all within-scanner repeat sessions) was acquired with version 5.9.0.0. The majority (78%) of scan sessions were completed by the same eight radiographers, four in Nottingham and four in Oxford. The remaining 22% of scan sessions were completed by another group of eight radiographers. Both scanner software version and radiographer per scan session are provided as part of the repository.

All data were acquired after informed consent was obtained from all participants. All participants consented to their data being shared with other researchers, including via online repositories, as long as they are shared in a form that does not identify them. Full details were provided in the Participant Information Sheet on data anonymisation for demographics (age, sex) and “defacing” of high-resolution anatomical scans. Ethical approval was obtained by the University of Nottingham Faculty of Medicine and Health Sciences Research Ethics Committee (FMHS-REC 452-0122) and the protocol was also reviewed and approved by the University of Oxford Medical Sciences Research Ethics Committee (R79669/RE001). For some earlier data of Phase A, ethical approval was obtained by the University of Nottingham Faculty of Medicine and Health Sciences Research Ethics Committee (FMHS-H14082014/47), also following an agreed technical development protocol (FMRIB_004_V4) approved by the University of Oxford Research Ethics Service and Oxford University Clinical Trials and Research Governance office, with explicit consent on data sharing of anonymised data, as described above.

### Data preprocessing

**Fig. 4:**
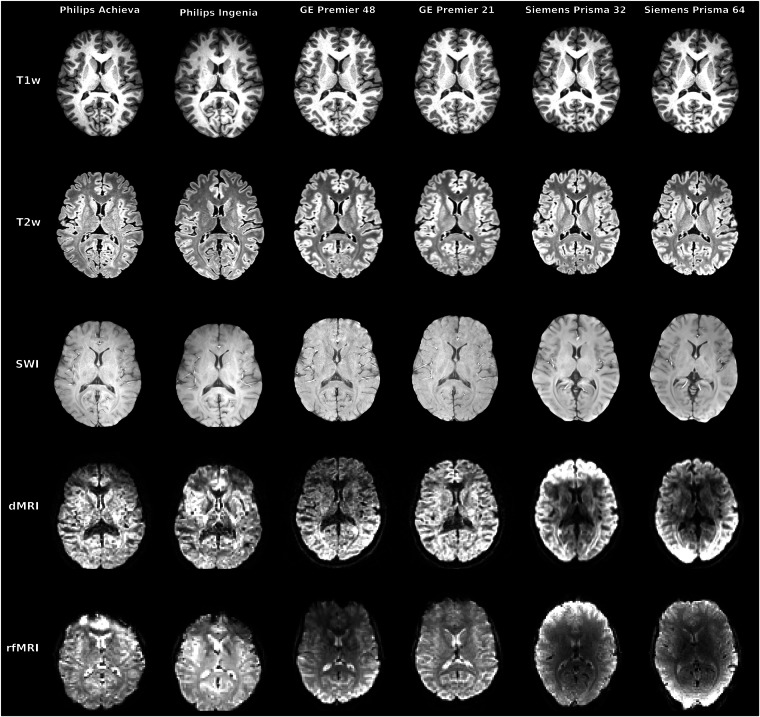
Qualitative examples of all modalities for a single participant data across all scanners. Raw acquired data are presented, without any preprocessing (other than skull-stripping) or registration performed. For fMRI, the first volume is shown. For dMRI, a single *b* = 1000 *s/mm*^2^ is shown corresponding to a left-right diffusion-sensitising orientation.

Data processing follows that previously used for Phase A^9^. Data DICOMs were converted to NIfTI format using dcm2niix (v1.0.20211006)^45^ and subsequently converted to the BIDS data structure^37^. All data have been anonymised and the high resolution anatomical images have been “defaced” following the UKBB pipeline defacing procedures^36^. Anonymised and defaced BIDS format data are available through the OpenNeuro repository (https://openneuro.org/datasets/ds004712)^38^. Examples of the acquired data for a single participant’s complete acquisition (all modalities and scanners) are shown in Fig. 4.

Raw data were pre-processed and thousands of imaging-derived phenotypes (IDPs) were extracted using a modified version (https://github.com/SPMIC-UoN/ON-Harmony_UKBB_pipeline/tree/ON-Harmony) of the UK Biobank pipeline^36^. Modifications are described in detail in^9^ and were necessary to accommodate onboarding of data from different vendors. In addition, we extracted cortical parcellations using FreeSurfer^46^. In summary, main categories of IDPs per modality include: a) For T1w and T2w, brain tissue volumes including tissue (white matter, grey matter, cerebrospinal fluid) segmentations, cortical and subcortical segmentations, and region-wise cortical surface area, mean thickness, mean curvature and volume derived from FreeSurfer^46^ for two cortical parcellations; b) For dMRI, regional (atlas-based, skeletonised white matter ROIs) and tract-wise (tractography-derived tract masks) mean microstructure features (DTI: FA, MD, MO, L1, L2, L3, NODDI: ICVF, ISOVF, ODI); c) For rfMRI, and for nodes derived from a 25-dimensional group ICA, full and partial correlation pair-wise functional connectivity values and node amplitudes, d) For SWI, T2* estimated for seven bilateral subcortical structures.

**Fig. 5:**
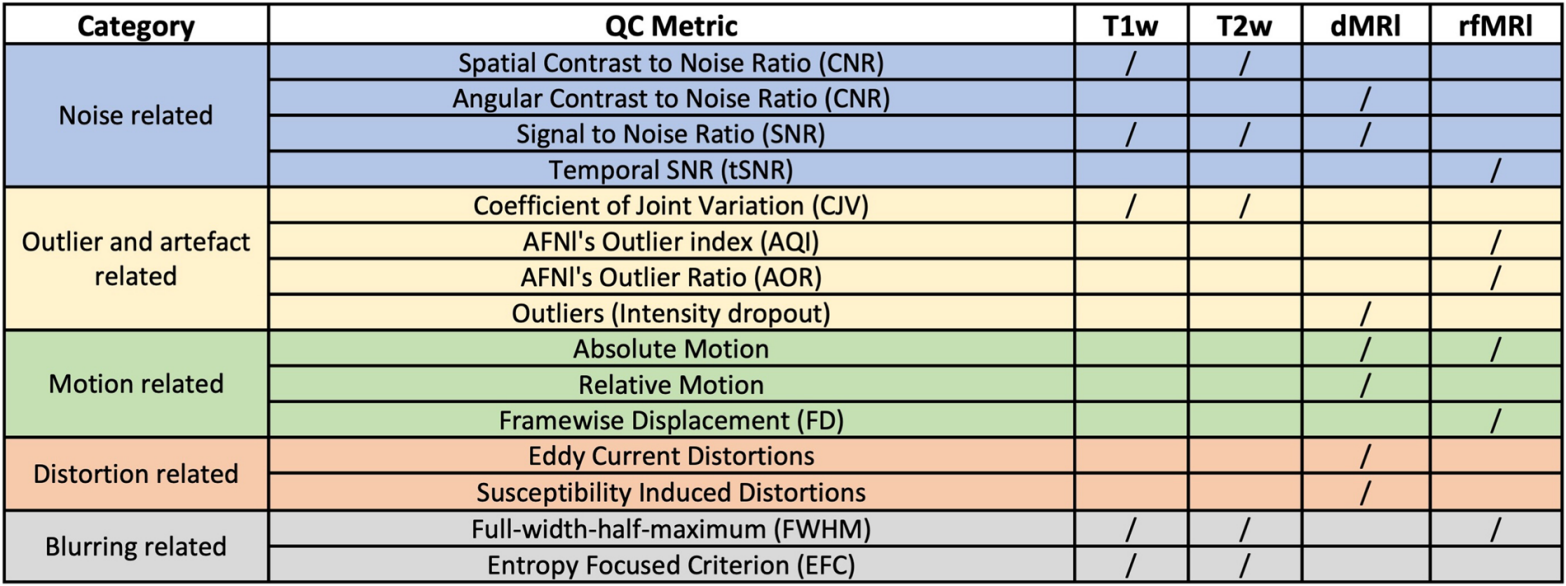
The list of image quality metrics (IQMs) for each modality derived using MRIQC for T1w, T2w and fMRI, and eddyQC for dMRI. ‘/’ denotes the modality for which the metric is used. Reproduced and modified from^9^.

In addition to the IDPs, we generated image quality metrics (IQMs) for each scan session. We derived IQMs for T1w, T2w and rfMRI using MRIQC (v22.0.6, https://hub.docker.com/r/nipreps/mriqc/)^47^, and for dMRI using eddyQC^48^. Extracted IQMs are summarised in Fig. 5. We provide a data dictionary describing derived IQMs, IDPs and session information OpenNeuro release^38^.

All processing was performed using a CentOS Linux 7 system with dual GPU (NVIDIA Tesla K80) acceleration for several aspects of the pipeline. Extraction of imaging features across all sessions allowed us to obtain summary measures of IDP variability of the same participant across different scanners and across within-scanner repeats, as well as characterise the variability of a given IDP across participants.

## Data Records

The OpenNeuro database (https://openneuro.org/datasets/ds004712)^38^ contains both imaging and non-imaging data. In terms of MRI, the database contains NIfTI images that have been anonymised and high-resolution anatomical scans (T1w and T2w) have been “defaced”; to avoid rendering of the face, facial features including ear, nose and eyes have been removed. Each participant has a dedicated folder containing subfolders for each of the corresponding scanning sessions (six sessions for each participant and 11 sessions for those participants that had within-scanner repeats). For each scanning session, a corresponding directory follows the following convention: sub- < ParticipantID > _ses- < ScanSite > < ScannerCode > < SessionNumber > format. For instance sub-15320_ses-NOT1ACH001, indicates the scanning session for participant 15320, scanned in the Nottingham Philips Achieva for the first time. Scan site and scanner codes are NOT1ACH, NOT2ING, NOT3GEM, NOT4GEP, OXF1PRI, OXF2PRI, OXF3TRI, OXF4GEP indicating respectively the Nottingham Philips Achieva, Nottingham Philips Ingenia, Nottingham GE MR750, Nottingham GE Premier (48 channel), Oxford Siemens Prisma (32 channel), Oxford Siemens Prisma (64 channel), Oxford Siemens Trio, and Oxford GE Premier (21 channel). In total, the database is approximately 58 GB.

Imaging data were all converted and are released into the BIDS standard format^37^, which is a commonly used neuroimaging data format designed for consistent and simplified data organisation, and hence data sharing. Upon uploading data to OpenNeuro, the data are BIDS validated to ensure that they meet BIDS requirements. Within each session folder, the data is categorized into anatomical (“anat”), diffusion (“dwi”), fieldmap (“fmap”), functional (“func”), and susceptibility-weighted (“swi”) imaging. The primary NIfTI data are each accompanied by corresponding JSON sidecar files. For diffusion data, additional files containing b-vectors and b-values are included, which indicate the diffusion-sensitising gradient directions and amplitudes. As the anatomical data are defaced, defacing NIfTI mask files (i.e. a binary image file used to remove facial features) are also provided for each session.

In addition to imaging data, the root folder contains non-imaging data organised in several files. The ‘participants.tsv’ file, described in the accompanying ‘participants.json’, provides a summary of each participant’s sex, age at first scan, and study phase (A or B). It has 20 rows, one per participant. The ‘session_delays.csv’ provides for each scanning session the delay (in days) between the participant’s first scanning session and the particular session. First sessions for a participant are encoded as having a “0” delay. The information is organised as a participants (20 rows) by session (13 columns) matrix. As not all participants have within-scanner repeats, columns titled “repeat_*” are left blank for these participants. In addition, as the scanners changed between study phases, the GE Premier columns are left blank for Phase A and the GE MR750 and Siemens Trio columns are left blank for Phase B. For clarity, we include additional columns denoting the phase for each participant and the scanner used for within-scanner repeats. The ‘session_radiographers.csv’ provides the radiographer (coded as RG1, RG2, etc…) for each session. It is organised as a participants by sessions matrix as in ‘session_delays.csv’. The ‘software_versions.csv’ provides the scanner software version for each session (again organised as a participants by sessions matrix). Finally, the ‘protocol_accelerations.csv’ provides the acquisition accelerations, effective echo spacing (EES) and total readout time (TRT) for fMRI and dMRI data for each scanner. These are organised as two scanner by parameter matrices (one matrix for each modality), following the structure of Fig. 3.

The repository also contains imaging-derived IQMs and IDPs in CSV format, ‘all_idps.csv’. These are organised as *N × M* matrices, where *N = *165 is the number of scanning sessions and *M = *2,513 is the number of IDPs/IQMs. The first 100 columns of this CSV contain session information, including scanner and participant information, and IQM metrics. The ‘data_dictionary.pdf’ file includes a detailed description of all columns included in ‘all_idps.csv’. The ‘dataset_description.json’ file includes essential information about acknowledgments, funding, and ethical approval.

The data are accompanied by a ‘README’ file, which provides general details on participants, scanners, incidental findings, and any deviations from protocols. The incidental findings process was raised for two of the participants, but in both cases they were deemed non-pathological by consultant radiologists. Another text file describes acquisition protocol deviations. While the majority of participants were scanned using consistent protocols, there were minor variations in protocol parameters for some sessions, due to radiographer or image reconstruction errors. The deviations are all described in the corresponding file.

## Technical Validation

Data from all sessions and a representative set of derived maps were first inspected for discrepancies and artifacts. Qualitative examples of raw and preprocessed data showcasing different steps of the pipeline are presented in Supplementary Videos 1–3. Rows correspond to different participants and columns to different scanners. Supplementary Video 1 shows the anatomical T1w images in native space and how they are warped to MNI-152 standard space after non-linear registration. Supplementary Videos 2, 3 show the original EPI data followed by the data fieldmap-corrected for susceptibility-induced distortions, for dMRI and rfMRI respectively.

In the following sections, a number of further assessments are presented to quantitatively demonstrate the quality of the acquired data and the breadth of information conveyed. Firstly, we present the image quality metrics (IQMs), across modalities, participants and scanners. Secondly, we map different pools of variability for the hundreds of multi-modal imaging-derived phenotypes (IDPs) to validate that data acquired from the same participant in the same scanner are less variable than those acquired from the participant between different scanners. Thirdly, we showcase the reuse potential of the resource and confirm that the ON-Harmony resource can provide insight into the efficacy of explicit harmonisation tools, like ComBat^20^; and into the generalisability across scanners/vendors of neuroimaging processing tools, like FSL-XTRACT^49^ (implicit harmonisation). In doing so, we validate the quality and breadth of the data by revealing expected trends.

### Image quality metrics (IQMs)

**Fig. 6:**
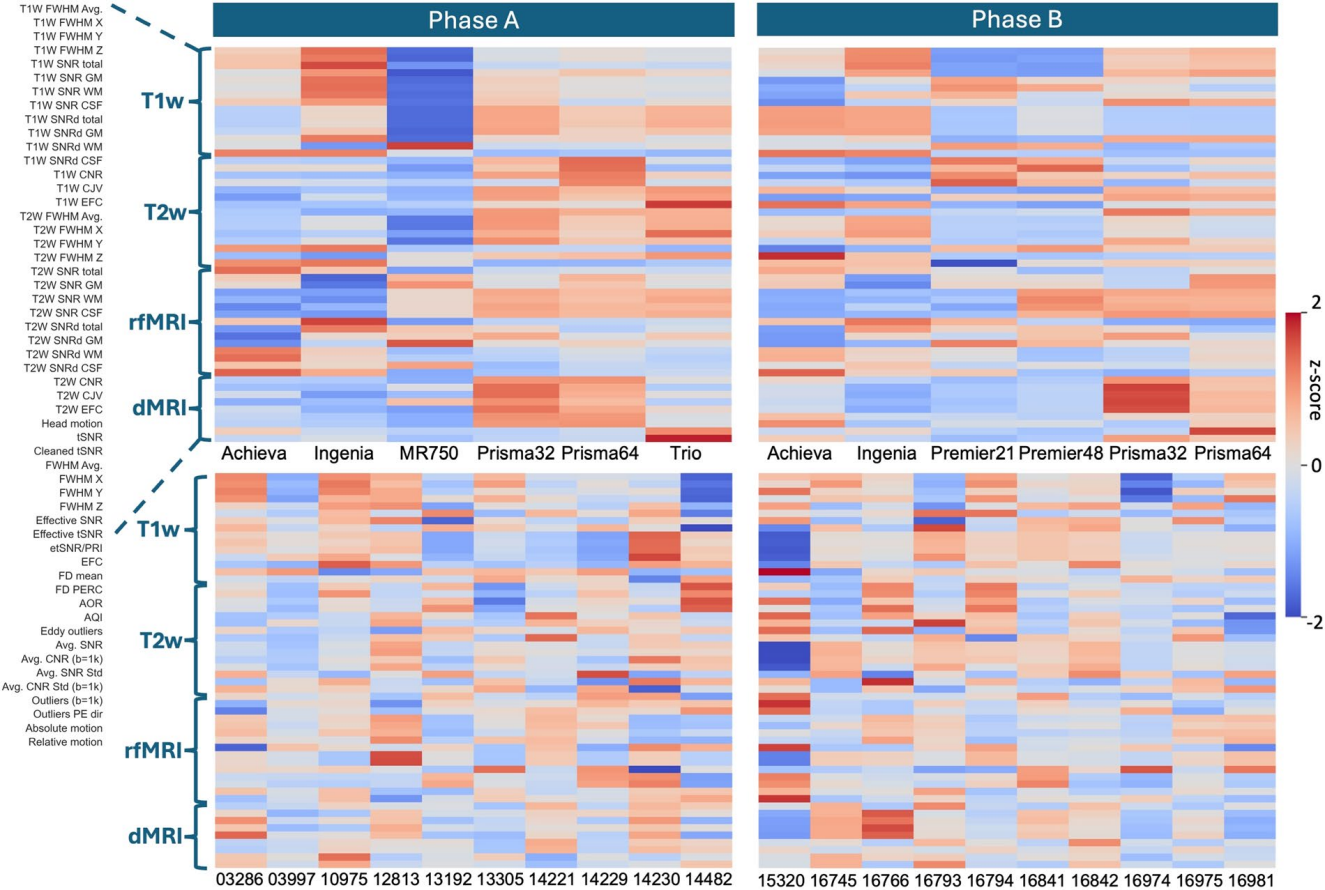
Heatmaps of the variability of image quality metrics (IQMs) for T1w, T2w, rfMRI and dMRI data for each phase. Top row: IQM variability across scanners is calculated by z-scoring individual IQMs across scanners and averaging across participants. Bottom row: IQM variability across participants is calculated by z-scoring individual IQMs across participants and averaging across scanners. Within-scanner repeats are excluded.

IQMs were extracted for each scanning session and reflected signal-to-noise-ratio (SNR), contract-to-noise-ratio (CNR), imaging artifacts and distortions across modalities, and participant motion, as described in Fig. 5. The metrics were z-scored across participants or scanners, to identify particular participants or scanners that deviated considerably from the average. Specifically, to assess quality across scanners, for each participant the IQMs across scanners were z-scored (i.e. each IQM had the mean IQM value across scanners for that participant subtracted and divided by the corresponding standard deviation) and then the resultant z-scored maps were averaged across participants. To assess image quality across participants, for each scanner the IQMs were z-scored across participants and then the z-score maps were averaged across scanners. These maps provided an indication of how each IQM differed from the corresponding mean with respect to other scanners/participants in the data, allowing us to identify potential outliers. Figure 6 shows heatmaps of these z-scores for both phases of the study. As observed, although there are some deviations from the mean for certain participants or scanners, there are no significant outliers, as the absolute values of the z-scores do not exceed 2.12. For Phase A, 85.74% of IQMs for participants and 74.4% of IQMs for scanners are within one standard deviation of the respective mean and 99.81% of participant IQMs and 100% of scanner IQMs are within two standard deviations. For Phase B, 85.74% of IQMs for participants and 80.25% of IQMs for scanners are within one standard deviation of the respective mean and 99.26% of participant IQMs and 100% of scanner IQMs are within two standard deviations. Nevertheless some useful trends are shown, for instance the Prisma32 has the highest SNR/CNR for diffusion and the wide-bore Ingenia with the lowest gradient strength has the lowest SNR/CNR. The MR750 has the highest T1w and T2w IQM deviations, as a non-MPRAGE sequence was used for the T1w and a 2D sequence was used for the T2w-FLAIR (as opposed to a 3D sequence for the other scanners).

### Imaging derived phenotypes (IDPs)

As presented in Methods, we computed hundreds of IDPs across all modalities. We mapped three modes of variability for each of the IDPs to ensure derived features follow expected trends. Specifically, we mapped the variability of IDPs across scanners, depicting how imaging features of the same individual vary across different scanners. For reference, we also mapped variability in within-scanner repeats of the same individual, as well as between-participant variability for the same IDPs.

**Fig. 7:**
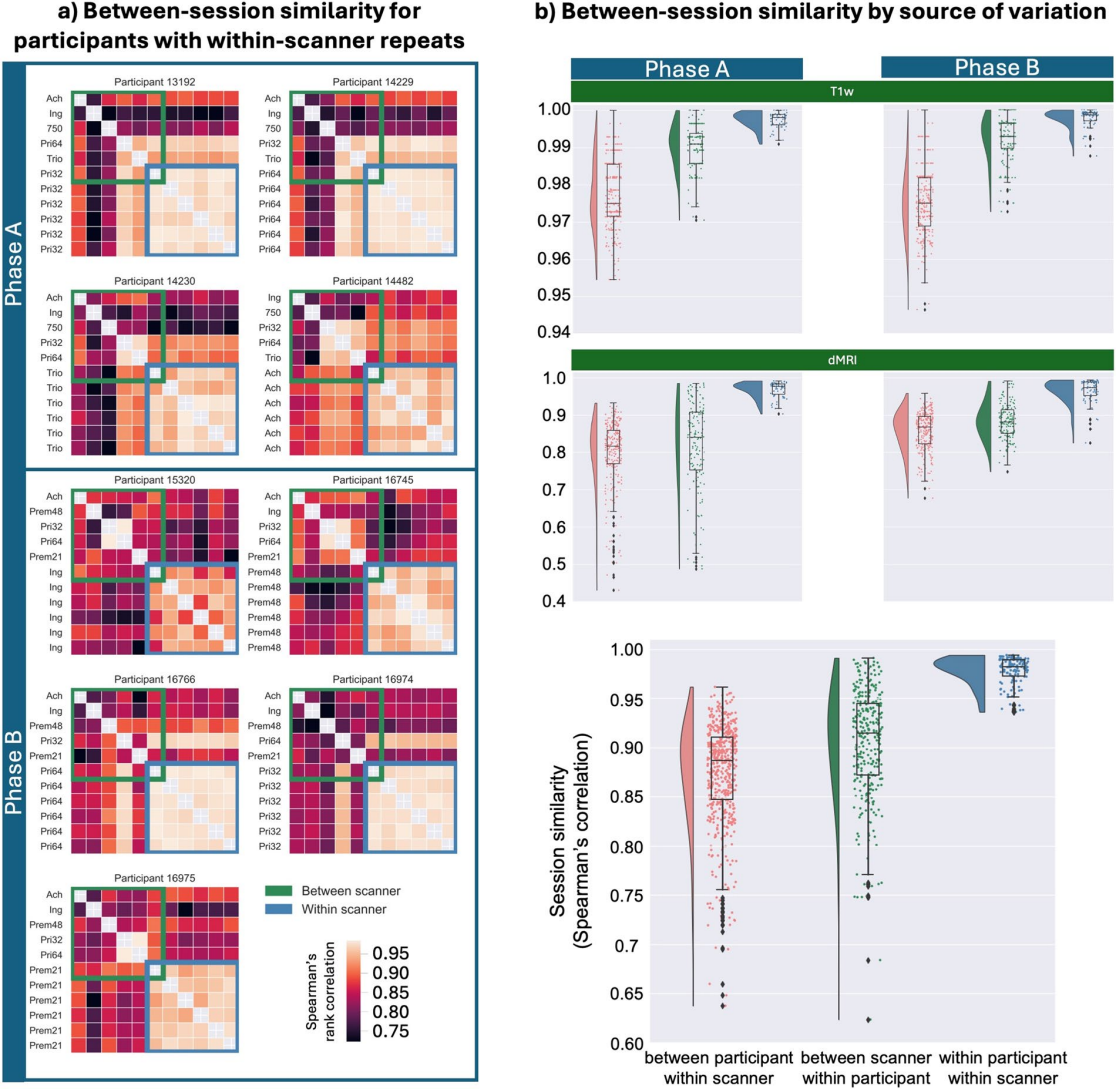
Variability of imaging-derived phenotypes (IDPs) across scan sessions. (**a**) Session-wise Spearman’s rank correlation matrices *P*_*ij*_ for all participants with within-scanner repeat sessions, averaged across *M* IDP groups. Within-scanner repeats are bound by the blue square and between-scanner repeats bound by the green square. IDP groups include subcortical volumes, brain tissue volumes, subcortical T2*, cortical parcel volumes, dMRI regional and tract-wise microstructure (FA, MD, MO, L1, L2, L3), and rfMRI pairwise functional connectivity and node amplitudes for a d = 25 cortical parcellation. (**b**) Session-wise Spearman’s rank correlation (proxy for “similarity”) distributions for between-participant within-scanner (pink), within-participant between-scanner (green) and within-scanner within-participant (blue) pools quantified for each phase and for two example modalities (T1w - top plots, dMRI - middle plots), and across all IDP-groups and across phases (bottom plot).

Figure 7 maps these pools of variability in different ways. Panel (a) shows between-session similarity of IDPs for each of the nine participants that had within-scanner repeats (i.e. *S = *11 scanning sessions in total per participant, where both between-scanner and within-scanner similarities are measured). High values correspond to pairs of scanning sessions whose IDPs are highly correlated. Specifically, for sessions *i* and *j* (1 ≤ *i, j* ≤ *S*) we used Spearman’s rank correlation to compare IDPs across session pairs. IDPs were grouped into *M* groups such that correlational analyses were performed in groups of IDPs with similar ranges of values to avoid bias. IDP groups included brain tissue volumes, subcortical T2*, cortical parcel volumes, dMRI regional and tract-wise microstructure (FA, MD, L1, L2, L3) and the top 5% strongest (identified by calculating the mean edge weight across within-scanner repeats for each of the participants that have within-scanner repeats) rfMRI functional connectivity values and node amplitudes for a 25-node cortical parcellation. The session similarity value *P*_*ij*_ for a single participant is represented by the median across the IDP groups, i.e.: *P*_*ij*_ = *median*(*rcorr*(${f}_{i}^{m}$, ${f}_{j}^{m}$)), with 1 ≤ *m* ≤ *M*; *rcorr* is the Spearman’s correlation function, *m* is the IDP group index, which spans across all the *M* IDP groups considered, and *f*_*i*_^*m*^ is the set of IDPs for session *i* and IDP group *m*; the median value, indicated by the angle brackets, is calculated over those IDP groups. For all participants we see that the IDPs for within-scanner repeats are more similar than ones for the between-scanner repeats. We also see some sub-groupings of the scanners that agree with expectation. For instance, sessions from the two Siemens Prisma scanners are more similar than the others, and the three narrow-bore, high-gradient scanners (two Prisma and the Achieva) also show higher similarities than the rest (for instance than the pair of the two wide-bore GE Premier scanners).

We grouped session-similarity values considering: within-scanner(-within-participant) repeats, between-scanner(-within-participant) sessions and between-participant(-within-scanner) sessions. Their distributions are reported in the violin plots in Fig. 7b in blue, green and pink respectively. Data are shown for phases A and B independently and for two exemplar modalities, as well as for both phases and modalities combined (bottom plot of Fig. 7b). In all plots, within-scanner similarity values are the highest and with the smallest spread compared to the corresponding between-scanner and between-participant distributions. For the individual modalities shown, T1w and dMRI, the between-scanner similarities improve from Phase A to Phase B, particularly for dMRI, probably due to the addition of two scanners with higher gradient specifications. However, in all cases the variation exhibited by the between-scanner distribution is not negligible compared to the variation in the between-subject case, hinting towards the need for harmonisation. This is more pronounced in certain modalities, such as dMRI and rfMRI, and less so in others, such as T1w.

**Fig. 8:**
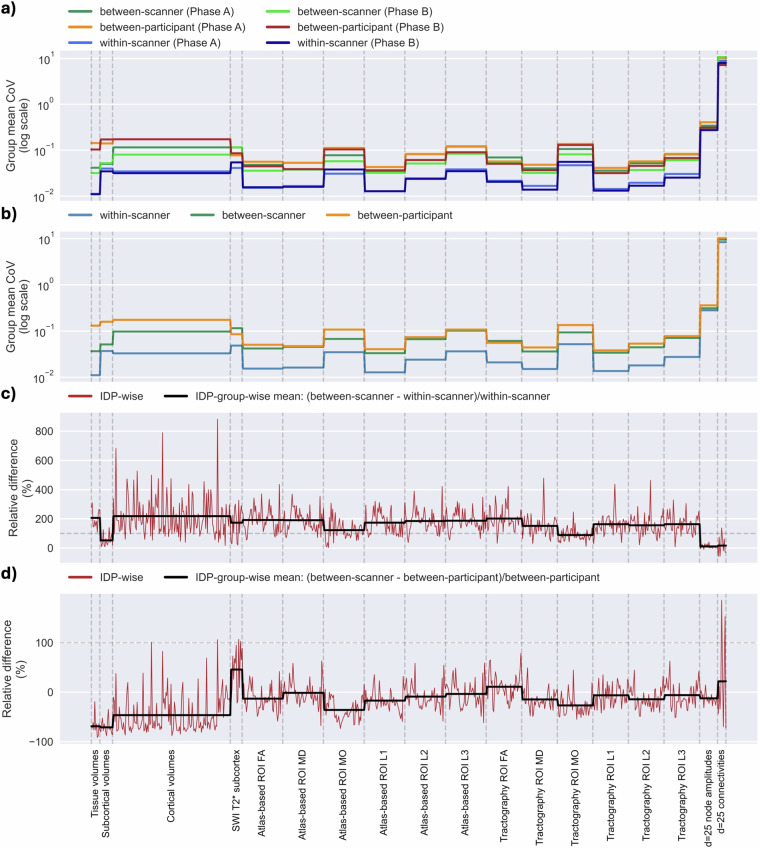
Variability of imaging-derived phenotypes (IDPs) between/within scanners and between participants estimated as the coefficient of variation (CoV). (**a**) IDP-wise CoV between-scanners (greens), within-scanner (blues) and between-participants (orange/red) is calculated for each phase. For ease of visualisation, we present the IDP-group averaged CoV and using a log-scale. (**b**) IDP-group averaged CoVs (log-scale) combining phases for within-scanner (blue), between-scanner (green) and between-participant (orange). (**c**) the relative difference (in percent) between the between-scanner and within-scanner CoV, plotted at the IDP and IDP-group levels. (**d**) as in c) but for the relative difference between the between-scanner and between-participant CoVs. See main text for more details.

To validate consistency of IDP variability trends in the two independent phases A and B, as well as the full cohort, we mapped variability for individual IDPs as the coefficient of variation (CoV) across between-scanner repeats of a participant (i.e. between-scanner, within-participant) against baseline sources of variability: (i) the CoV of within-scanner, within-participant repeats, (ii) the CoV of within-scanner, between-participant (i.e. proxy of biological variability) repeats. Figure 8 plots these pools of variability for each phase independently and combined. For simplicity of visualisation, we plot the IDP group-averaged CoV. Figure 8a shows the IDP-group averaged between-scanner within-participant (green tones), within-scanner within-participant (blue tones) and between-participant within-scanner (orange/red tones) CoVs for phases A and B independently, showing overall agreement in trends across phases although with some reductions observed in Phase B. Panel (b) shows the within-scanner between-participant (blue), between-scanner within-participant (green) and between-participant within-scanner (orange) IDP group-averaged CoVs for all data across both phases. These are then compared in panels (c-d). In panel (c), we compare the between-scanner within-participant and within-scanner within-participant sources of variability by taking their relative difference. Relative difference is calculated at the scanner/participant level and average across scanners/participants is reported. We calculated the IDP-wise (red) relative differences and subsequently the IDP group-averaged (black) and find that, in almost all cases, the between-scanner within-participant variability is greater than the within-scanner within-participant variability as one would expect. In panel (d), the between-scanner within-participant variability is compared to the between-participant within-scanner variability as above. In this case, we observe that between-scanner within-participant variability can be as large and occasionally exceed between-participant within-scanner variability for a number of IDP groups, in agreement with what has been reported before^10,50,51^, highlighting the need for harmonisation in between-scanner data.

### Efficacy of explicit harmonisation with ON-Harmony

**Fig. 9:**
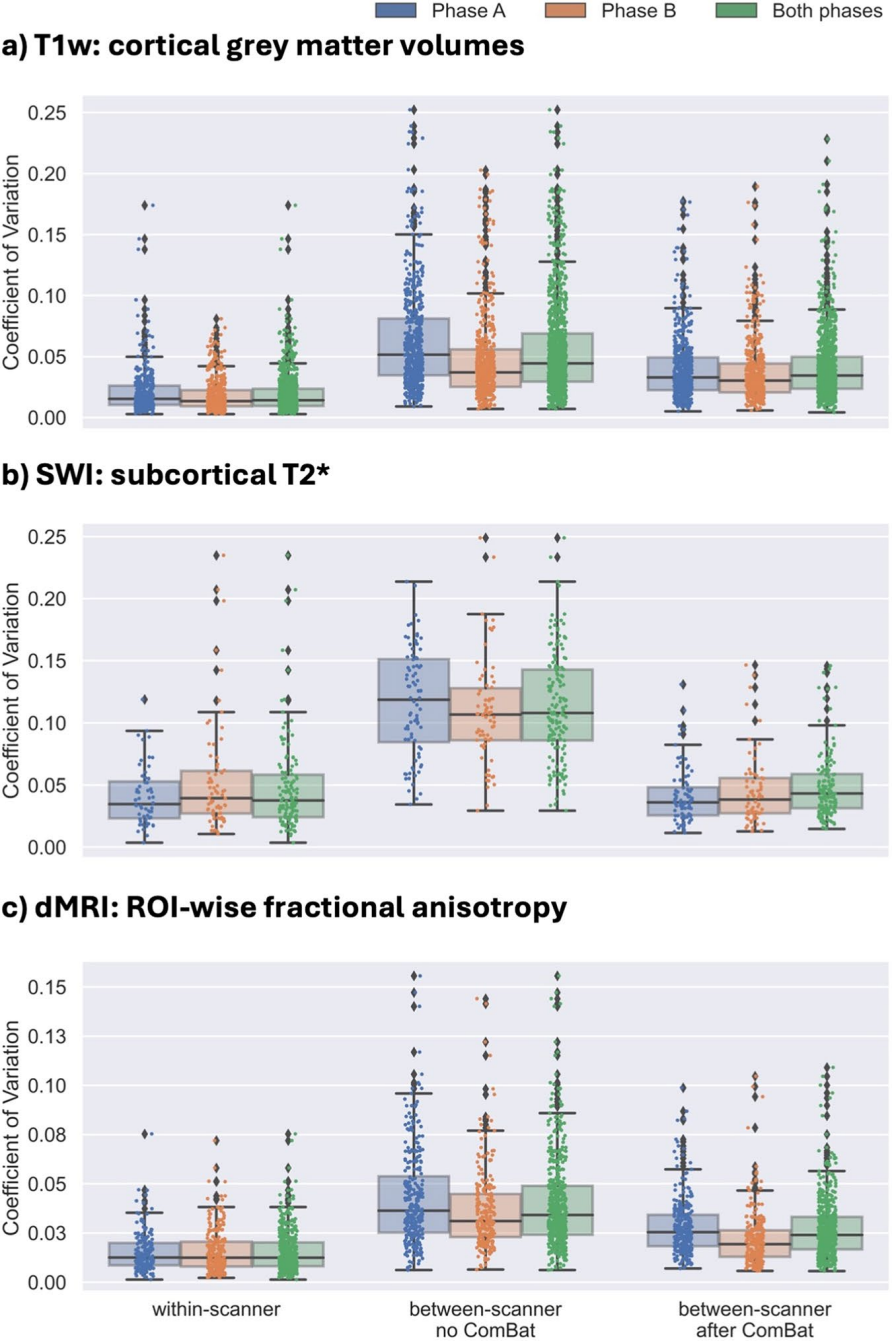
Efficacy of a commonly used explicit-harmonisation approach (ComBat) with ON-Harmony. The within-scanner and between-scanner (before and after harmonisation with ComBat) CoVs are calculated for three exemplar IDP-groups: (**a**) atlas-based cortical grey matter volumes derived from T1w, (**b**) white matter microstructure (fractional anisotropy) for regions of interest derived from dMRI, and (**c**) estimates of subcortical T2* from SWI. This is repeated separately for each phase (Phase A: blue, Phase B: orange) and after combining phases (green).

Given the differences in between-scanner vs within-scanner variability, we validated that a commonly used explicit harmonisation algorithm mitigates such differences in independent data from Phase A and Phase B, as well as in the full cohort. ComBat^20^ is typically used to estimate batch effects on IDPs (i.e. non-biological nuisance factors) and remove them from imaging features. Figure 9 shows how between-scanner variability in ComBat-processed IDPs is reduced compared to pre-ComBat between-scanner variability and within-scanner variability for the same IDPs. Specifically, variability pre and post-ComBat was quantified for three representative groups of IDPs: atlas-based cortical grey matter volumes, region-wise white matter skeletonised fractional anisotropy (FA), and subcortical T2* from SWI. The CoV was quantified for within-scanner within-participant repeats, between-scanner within-participant repeats (pre-ComBat) and post-ComBat between-scanner within-participant repeats. ComBat (v0.2.12, https://github.com/Jfortin1/neuroCombat)^20^ was applied to each IDP group separately and by treating each phase as an independent cohort and after combining data: 1) including data from Phase A only, 2) including data from Phase B only, 3) including data from both phases. As standard, participant age and sex were used as covariates. In each IDP group, and for each case, ComBat effectively reduced the between-scanner within-participant variability as expected, with comparable performance across phases. The post-ComBat variability was higher than, but towards the within-scanner within-participant variability baseline. This use case further highlights how the presence of both within and between-scanner data in the ON-Harmony resource can be beneficial in evaluating the efficacy of explicit harmonisation approaches in an objective manner.

### Generalisability of processing across vendors (implicit harmonisation) with ON-Harmony

**Fig. 10:**
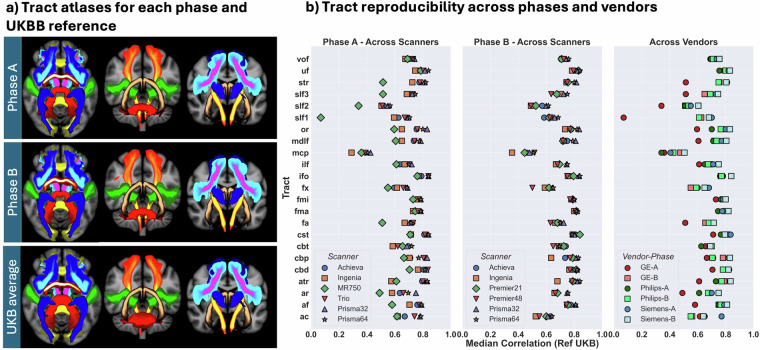
Generalisability of a commonly used processing tool (XTRACT) with ON-Harmony. (**a**) white matter fibre bundle atlases using XTRACT data from Phase A, Phase B, and a UK Biobank-derived atlas. Qualitative assessment of 42 WM fibre bundles considered shows good agreement between phases and the atlas. (**b**) Median correlation between each white matter fibre bundle reconstruction of the ON-Harmony sessions and the reference atlas (after applying a probability threshold of 0.1%, within the atlas-derived fiber bundle mask). Calculated per scanner separately for each phase of the ON-Harmony data (panels 1 and 2). Also calculated per vendor and compared between phases (panel 3). The UK Biobank atlas is an average of 100 unrelated participants.

The ON-Harmony data and IDPs can provide insight into generalisability of processing tools across scanners/vendors. This is important, as such generalisability can offer implicit harmonisation, i.e. processing pipelines that are less susceptible to scanner effects. Figure 10 shows an example by mapping results of white matter tractography (which forms the basis for a number of dMRI IDPs in the ON-Harmony repository) across scanners and vendors. Specifically, the figure shows results from FSL-XTRACT^49^, which maps a range of white matter bundles, as part of the data preprocessing and IDP generation.

The average white matter fibre bundle reconstructions for each phase are shown in Fig. 10a, along with a reference atlas obtained from UK Biobank data^35^ (100 participant average), qualitatively confirming high similarity in resultant tract reconstructions. For each tract, the average for a given scanner across all participants was obtained and their correlation against the reference atlas is shown in Fig. 10b. This shows moderate to high correlation values across all scanners with generally consistent trends across scanners and phases, although with some exceptions. For instance, correlation values for the MR750 are, on average, reduced compared to the other scanners with some white matter fibre bundles (superior longitudinal fasciculi SLFs) showing notable reductions in performance. This is likely due to the limited capability of resolving complex fibre crossings with the single shell data and the limited number of diffusion directions acquired using the MR750. In addition, for all scanners, the middle cerebellar peduncle (MCP) seems to be an outlier, with a much lower correlation than the rest of the tracts. This reflects differences in the field of view covering the cerebellum, as preference was given for all scanning protocols to fully cover cortex, resulting in non-optimal coverage of the cerebellum for scanners where fewer slices could be acquired within the available scanning slot. As expected, an improvement in reconstruction performance can be seen when comparing results from the GE MR750 to the GE Premiers in Phase B. This reflects the enhanced richness of the GE dMRI data in Phase B (multi-band, multi-shell) compared to Phase A (single-band, single-shell). These results showcase another potential use case of the ON-Harmony data for assessing cross-scanner/vendor generalisability for neuroimaging processing tools.

## Supplementary information

Supplementary video

Supplementary video

Supplementary video

## Data Availability

The adapted UKBB pipeline used is available via GitHub (https://github.com/SPMIC-UoN/ON-Harmony_UKBB_pipeline/tree/ONHarmony). All analyses were performed in Python 3.10.9. Data were handled using numpy 1.21.6 and pandas 1.5.3. Plots were generated using matplotlib 3.7.0 and seaborn v0.11.0. Statistical analyses were performed using pandas and scipy 1.10.0. Python scripts used for analyses are available on GitHub (https://github.com/SPMIC-UoN/ON-Harmony). All software used are freely available.
