# Supplementary figures and images for: A multi-site, multi-modal travelling-heads resource for brain MRI harmonisation
Source: Sci Data. 2025 Apr 11;12:609. doi: 10.1038/s41597-025-04822-2 (PMC11992253; doi:10.1038/s41597-025-04822-2)

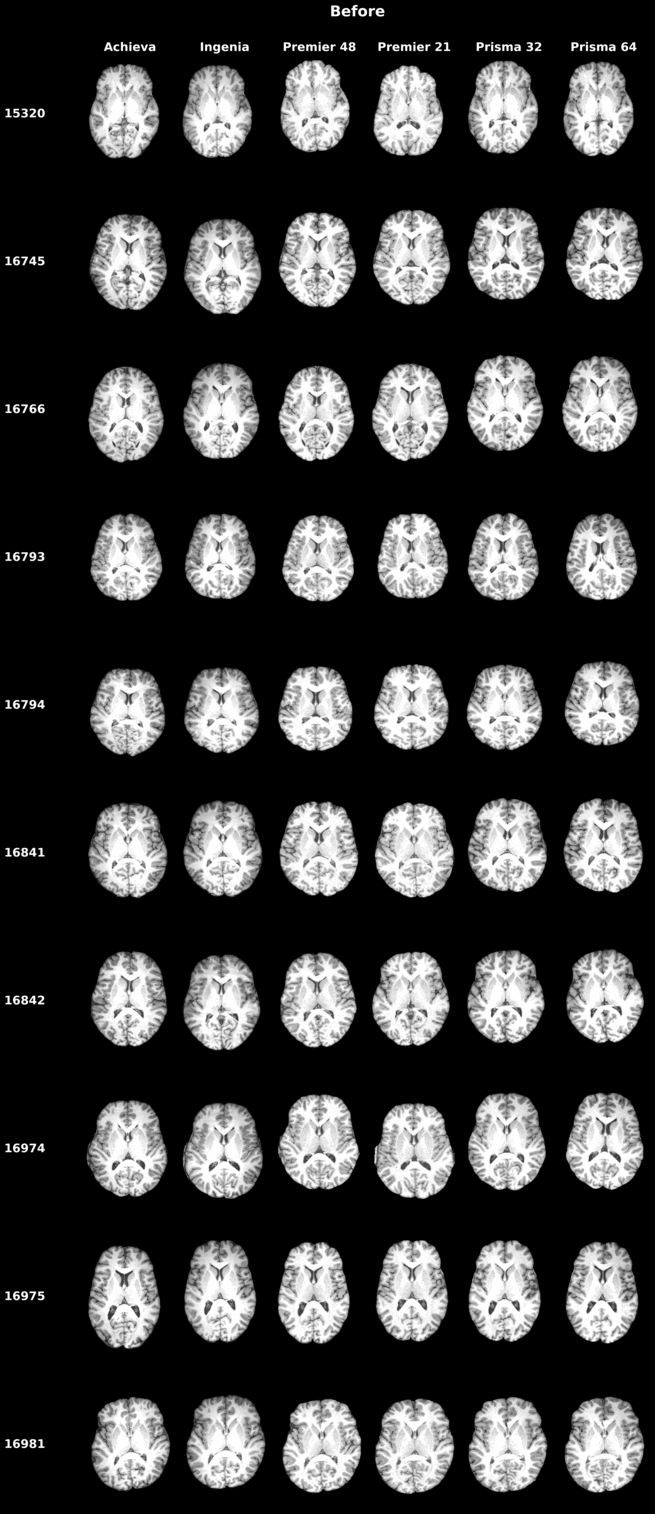

Supplement: Supplementary file 1 — Supplementary video [file 41597_2025_4822_MOESM1_ESM.gif]

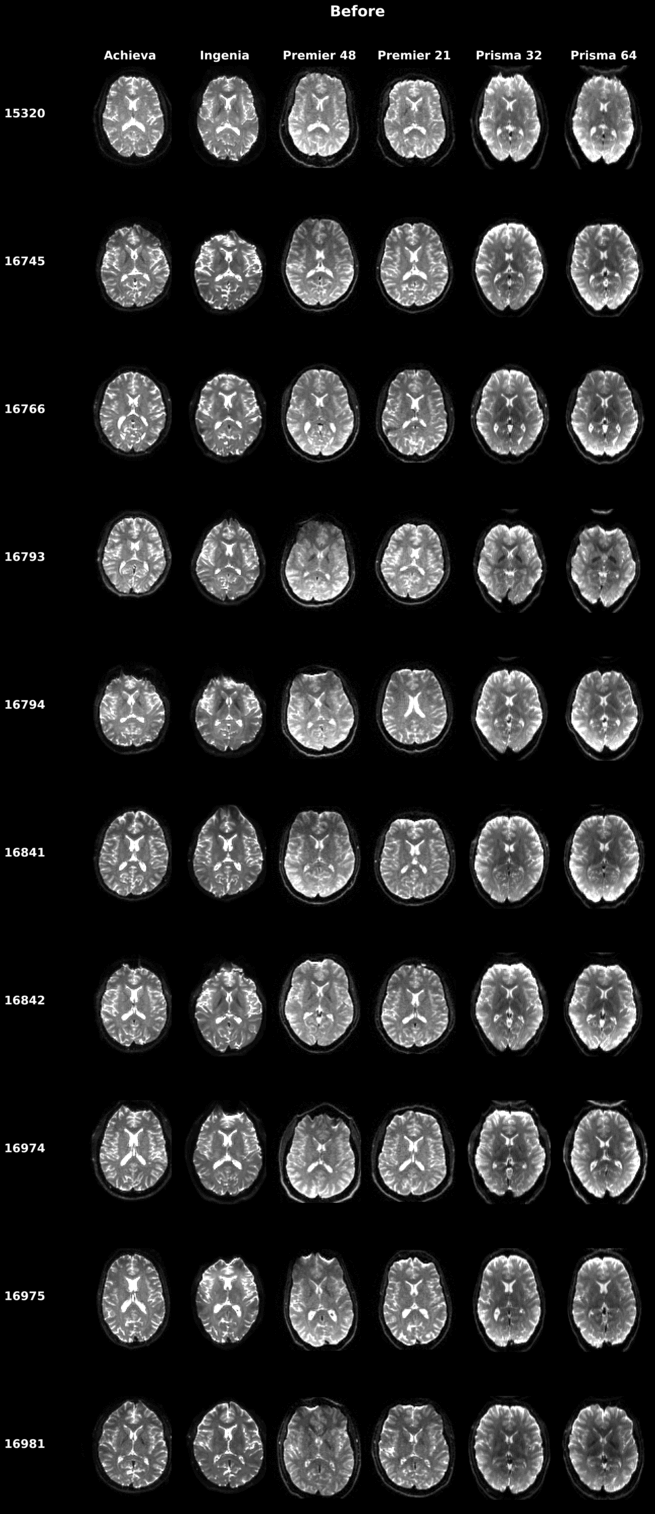

Supplement: Supplementary file 2 — Supplementary video [file 41597_2025_4822_MOESM2_ESM.gif]

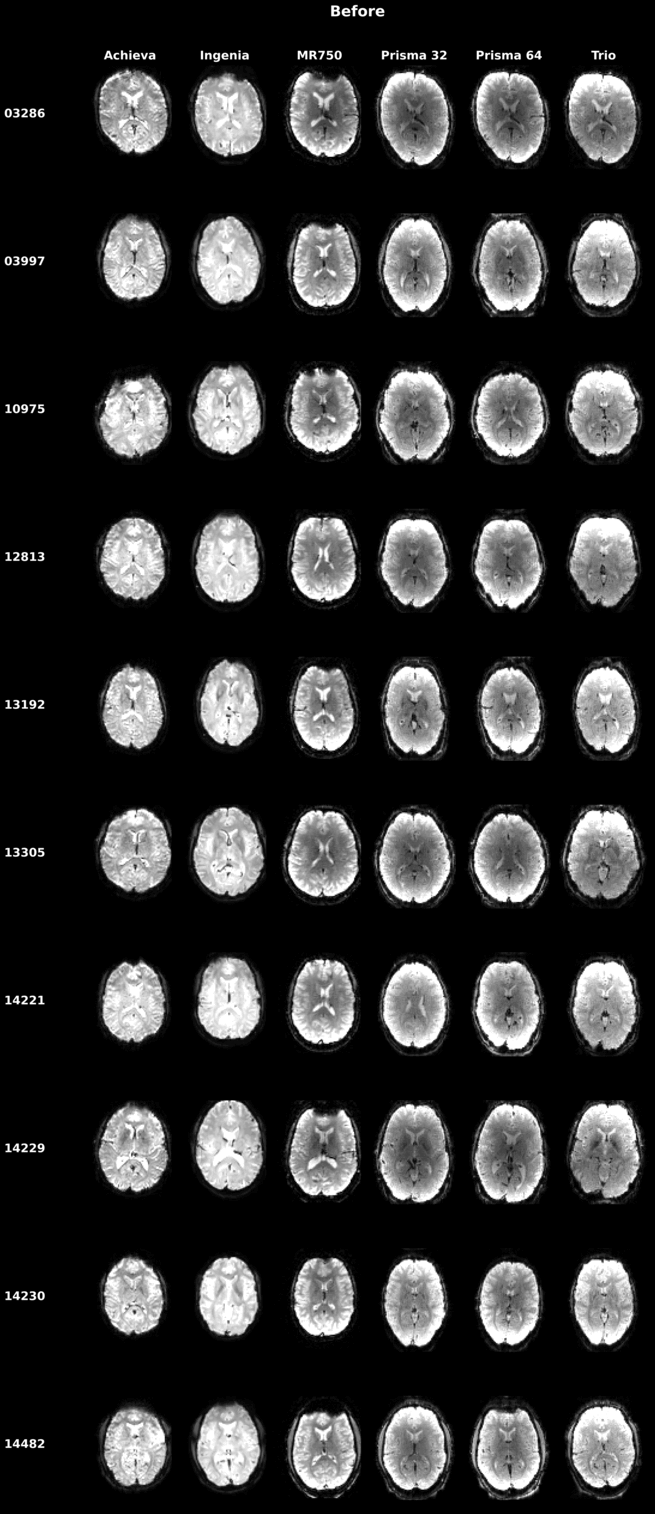

Supplement: Supplementary file 3 — Supplementary video [file 41597_2025_4822_MOESM3_ESM.gif]
